# Natural Bioactive Compounds from Winery By-Products as Health Promoters: A Review

**DOI:** 10.3390/ijms150915638

**Published:** 2014-09-04

**Authors:** Ana Teixeira, Nieves Baenas, Raul Dominguez-Perles, Ana Barros, Eduardo Rosa, Diego A. Moreno, Cristina Garcia-Viguera

**Affiliations:** 1 Centre for the Research and Technology of Agro-Environmental and Biological Sciences, (CITAB), University of Trás-os-Montes and Alto Douro (UTAD), Quinta de Prados, 5000-801 Vila Real, Portugal; E-Mails: Anaisabelt90@gmail.com (A.T.); rdperles@utad.pt (R.D.-P.); abarros@utad.pt (A.B.); erosa@utad.pt (E.R.); 2 Phytochemistry Laboratory, Department of Food Science and Technology, National Council for Scientfic Research (CEBAS-CSIC), P.O. Box 164, Campus de Espinardo–Edificio 25, E-30100 Murcia, Spain; E-Mails: nbaenas@cebas.csic.es (N.B.); cgviguera@cebas.csic.es (C.G.-V.)

**Keywords:** *Vitis vinifera* L., grape pomace, phytochemicals, bioactivity, extraction, food industry, pharmaceutical, phenolic compounds

## Abstract

The relevance of food composition for human health has increased consumers’ interest in the consumption of fruits and vegetables, as well as foods enriched in bioactive compounds and nutraceuticals. This fact has led to a growing attention of suppliers on reuse of agro-industrial wastes rich in healthy plant ingredients. On this matter, grape has been pointed out as a rich source of bioactive compounds. Currently, up to 210 million tons of grapes (*Vitis vinifera* L.) are produced annually, being the 15% of the produced grapes addressed to the wine-making industry. This socio-economic activity generates a large amount of solid waste (up to 30%, *w*/*w* of the material used). Winery wastes include biodegradable solids namely stems, skins, and seeds. Bioactive compounds from winery by-products have disclosed interesting health promoting activities both *in vitro* and *in vivo*. This is a comprehensive review on the phytochemicals present in winery by-products, extraction techniques, industrial uses, and biological activities demonstrated by their bioactive compounds concerning potential for human health.

## 1. Introduction

Grape crops are one of the main extended agro economic activities in the world with more than 60 million tons produced globally every year. Thus, for example, 67 million tons of grapes were produced in 2012, with almost 23 million tons corresponding to European contributors [1]. This production is mainly addressed to fresh consumption as table fruit, juice, and raisins. In addition to these major uses, an important proportion of grape production is addressed to vinification processes, which constitutes a relevant traditional activity in several countries in Southwestern Europe (*i*.*e*., France, Greece, Italy, Portugal, and Spain), as wine is one of the most consumed alcoholic beverages in the world [2].

Wine production entails the generation of huge amounts of by-products mainly consisting in organic wastes, wastewater, emission of greenhouse gases, and inorganic residues [3]. After grape juice extraction the remaining pomace and stems are currently not valued as highly profitable waste, being mainly directed to composting or discarded in open areas potentially causing environmental problems [4]. Thus, the increasing demand for environment-friendly industrial production in addition to the challenge for gaining operational efficiency and minimizing by-product treatment cost in the wine industry has started to move this sector towards the adoption of preventative integrated waste approaches [5]. However, when prevention is not feasible, the development of innovative procedures to recycle, reuse, and recover these residues is consistent with the growing demand of green materials and renewable resources of nutrients and bioactive compounds for the feed/food, pharmaceutical, and cosmetic sectors, which will allow a reduced dependence on the current manufacturing activity with the starting raw materials. In this sense, the valorization of these wastes will provide further alternatives to reduce the environmental impact of winery activity.

The potential of the valorization of these agro-industrial by-products is currently supported by the extensive information available on their content of healthy promoting phytochemicals with valuable activity concerning to the prevention of oxidative reactions, cardiovascular problems disease, inflammatory processes, and degenerative pathophysiological state developed in adults [6,7,8].

The absolute amount as well as the relative proportion of these beneficial compounds in vinification residues is conditioned by a myriad of factors including genetic load of the separate grape varieties, agro-climatic conditions, fertilization procedures, and soil properties, among others. On the other hand, the specific wine-making processes as well as the time between the generation of waste and valorization activities, as well as the characteristics of the recycling and recover procedures have a direct impact on the final concentration of phenolic compounds in the material and, therefore, on the potential as a source of bioactive phytochemicals [9,10,11].

The secondary metabolites including phenolic acids, flavanols, flavonols, anthocyanins, and stilbenes [12], differ in their solubility and recovery yield, which complicates their individual or targeted extraction. In this sense, innovative and more efficient solvents and extraction methods such as high pressure and temperature extraction, supercritical fluids, or ultrasound and microwave assisted extractions have been reported in an attempt to enhance the efficiency of the extraction of phytochemicals from vinification residues [13,14,15,16].

The main objective of the present work is to compile a comprehensive update and review on the composition and functional aspects of winery by-products, providing detailed information about the valuable bioactive phytochemicals of these multipurpose materials. The technological advancements applicable for the potential recovery of valuable components, from wine-making industry wastes, have been also considered.

## 2. Main By-Products Derived from Winery Industry

The winemaking process is based on ancestral procedures, being more an art than a science. As a general rule, many artisanal practices are strongly rooted in the traditional vinification processes, as well as limited human resources or physical infrastructures during the production operations, limiting the update of the technological advances addressed to minimize waste production in several wine industries [3]. Therefore, implementation of waste management in the wine industry is a challenging task, making necessary the development of innovative and effective valorization procedures. In this sense, the growing demand of final products and the urgency of avoiding the environmental footprints of this agro-industrial activity have encouraged a tough legal framework to ensure the efficiency of the processes and to support the improvements of the recovery and recycling procedures.

The type of residues produced is closely dependent on the specific vinification procedures, which also affect the physic-chemical properties of the residual material, the characteristics of which determine its further use and specific valorization circuit in which it could be integrated. The major residues from wine-making activity are represented by: organic wastes (grape pomace, containing seeds, pulp and skins, grape stems, and grape leaves), wastewater, emission of greenhouse gases (CO_2_, volatile organic compounds, *etc*.), and inorganic wastes (diatomaceous earth, bentonite clay, and perlite) [17]. In this regard, it is estimated that in Europe alone, 14.5 million tons of grape by-products are produced annually [18].

The valorization of winemaking by-products is mainly represented by the elaboration of soil fertilizers as well as a fermentation substrate for biomass production and livestock feeds [19,20,21]. However, there are several constraints for current available options for reusing these unprofitable materials. For example, certain polyphenols present in winery by-products are known to be phytotoxic and display antimicrobial effects during composting, impairing their utilization for this purpose. Regarding their use in livestock feed, some animals show intolerance to certain components, such as condensed tannins, which negatively affect digestibility [22]. Hence, their valorization as a source of bioactive phytochemicals of application in pharmaceutical, cosmetic, and food industries might constitute an efficient, profitable, and environment-friendly alternative for residues [23].

### 2.1. Grape Pomace or Press Residues

Grape pomace is the winery waste originated during the production of must (grape juice) by pressing whole grapes. Currently, 9 million tons of this organic residue are produced per year in the world [10], which constitutes 20% (*w*/*w*), on average, of the total grapes used for wine production [24,25,26,27]. The reported differences on the relative proportion of grape pomace in comparison with the total amount of grapes used during the vinification process is due to the complete material considered as well as the incorporation or not of grape stems as part of the vinification residues when calculating the relative proportion [24,25,26,27].

Concerning the general composition of grape pomace, the moisture percentage varies from 50% to 72% depending on the grape variety considered and its ripening state. The insoluble residues from this material have a lignin content ranging from 16.8% to 24.2% and a protein content lower than 4%. In general, peptic substances are the main polymer-type constituent of the cell walls present in grape pomaces, ranging from 37% to 54% of cell wall posysaccharides. Cellulose is the second type of cell wall polysaccharides in abundance in grape pomaces, varying from 27% to 37% [28]. Due to this content in non-digestible polysaccharides, additional fermentation processes may be required to avoid gastrointestinal disturbances when this material is integrated within complex food/feed matrices as a form of valorization.

By its composition, grape pomace stands out out as a suitable material to be employed in different processes, in particular the extraction of grape seed oil and polyphenols (mainly anthocyanins, flavonols, flavanols, phenolic acids, and resveratrol), production of citric acid, methanol, ethanol, and xanthan via fermentation, and the production of energy by methanization. Additional uses addressed to obtain alcoholic drinks by short fermentations and distillation, have been also described for this vinification waste [29,30,31]. Based on its polyphenolic content, several studies have reported a high antioxidant activity of this by-product, suggesting the winery-derived grape pomace as an interesting source for natural antioxidants with application in pharmacological, cosmetic, and food industries [27,32,33].

When considering the separate fractions of grape pomace (seeds and peels), the relative proportion of seeds ranges from 38% to 52% of the dry material [34]. Additional reports have pointed out much lower proportion of seeds, which could be almost 15% of grape pomace [35,36]. On this matter, again technological and material management could be responsible for the final composition of this vinification residue. The information available on the composition of grape seeds (*w*/*w*) point out the content of up to 40% fiber, 16% essential oil, 11% protein, 7% complex phenolic compounds like tannins, and other substances like sugars and minerals [37]. Special attention has been paid to the (poly)phenolic content of grape seeds, ranging from 60% to 70% of total extractable compounds. This high concentration is of great interest, taking into consideration that during the pressing of grapes only a minor proportion of these compounds is extracted [38], and this fact has attracted the interest of the pharmaceutical, cosmetic, and food industry as a profitable source of natural antioxidants [39,40,41], such as MegaNatural^®^-Gold Grape Seed Extract by Polyphenolics, and Citricidal^®^ grapefruit seed extract by Nutribiotic.

Grape skins constitute 65% of the total material of grape pomace on average. Grape skin has been reported as a rich source of phenolic compounds, even though the final yield is dependent on the specific vinification process and the extraction method used (solvent, temperature, time, and other factors) [8,42,43].

The phytochemical profile of this agro-industrial by-product supports its use as an interesting source of bioactive phytochemicals and ingredients. Nevertheless, the lack of appropriate valorization processes makes it mainly used as compost or discarded in open areas potentially causing environmental problems. Research on extraction conditions and new designs are needed for optimization of the release of phenolics from grape skins to maximize the properties of the wine pomace [43].

### 2.2. Grape Stems

Grape clusters or grape stems constitute a residue of the winery industry partially applied as a source of astringent compounds, mainly represented by proanthocyanidins [44]. This material is removed before the vinification steps to avoid an excessive astringency of the wine or a negative effect on the organoleptic characteristics. Therefore, their consideration within the vinification process is not absolutely addressed, although this constitutes an indubitable residue produced by the winery industries.

The quantity of stems varies between 1.4% and 7.0% of the raw matter processed [45]. Currently, the commercial value of grape stems is low, being mainly used as animal feed or soil amendments. The scarce information available on grape stems composition indicates anyway that this could be a valuable and interesting source of dietetic fiber and antioxidants [23,46,47,48,49,50]. The average moisture percentage of grape stems has been reported as ranging from 55% to 80%, with the higher variability attributed to the grape variety. The content of grape stems alcoholic insoluble residues is 71%, on average, of the dry matter and no differences between red and white varieties have been observed [28].

Concerning the phenolic composition of grape stems, their content has been shown in flavan-3-ols, hydroxycinnamic acids, monomeric and oligomeric flavonols, and stilbenes [51,52,53]. In this regard, phenolics from grape stems have been shown to be approximately 5.8% on a dry weight basis [10].

The current knowledge on the nutritional and phytochemical composition of this plant material should encourage further research that contributes to a greater understanding of its composition and specific outcomes for its application in developing innovative added-value products.

### 2.3. Grape Leaves

To date, leaves from *Vitis vinifera* L. constitute the less studied or valorized residue of the grape crops and the winery industry. The scarce information available on the grapevine leaves composition informs on their content in organic acids, phenolic acids, flavonols, tannins, procyanidins, anthocyanins, lipids, enzymes, vitamins, carotenoids, terpenes, and reducing or non-reducing sugars [54,55,56,57,58]. The rich and varied chemical composition of these leaves has led to a considerable interest in this plant material as a promising source of compounds with nutritional properties and biological potential. Thus, grapevine leaves are employed in the production of food ingredients and its juice has been also recommended as an antiseptic for eyewash [59,60].

### 2.4. Wine Lees

Wine lees are the residues formed at the bottom of receptacles containing wine, after fermentation, during storage or after authorized treatments, as well as the residue obtained following the filtration or centrifugation of this product. Lees are mainly composed of microorganisms (mainly yeasts), tartaric acid, inorganic matter, and phenolic compounds [61]. Lees play a major role in wine processing as they interact with (poly)phenolic compounds, directly related to the colour and other organoleptic properties, and adsorb them [62]. Moreover, lees liberate enzymes favouring the hydrolysis and transformation of (poly)phenolic substrates in phenolics with high added-value and interest like gallic acid or ellagic acid [63]. The scarce literature on this issue has reported the presence of anthocyanins (6–11.7 mg·g^−1^·dw (dry weight)) and other phenolics (29.8 mg·g^−1^·dw) in wine lees [61], and Tao *et al*. described the concentration of total phenolic compounds in wine lees (50 mg·GAE·g^−1^·dw) [64].

## 3. Functional Compounds in By-Products from Organic Wineries

The well-established relationship between diet and health has prompted extensive research concerning the evaluation of the qualitative and quantitative content of bioactive phytochemicals in plant foods and generated side streams (*i*.*e*., harvest remains, non-marketable produce, by-products, *etc*.). These bioactive compounds are secondary metabolites produced by plants under stress conditions such as wounding, infections, and UV irradiation [65,66], and thus these compounds constitute a physiological tool of plants used to maintain health status. Bioactive phytochemicals present in winery by-products are mainly represented by (poly)phenols, which arise biogenetically from two main primary biosynthetic pathways: the shikimate and acetate pathways. These compounds are structurally comprised of one or more aromatic rings bound to distinct moieties. Thus, their chemical structures include a range from simple molecules, such as phenolic acids, to complex polymeric structures like tannins [10,67]. Additionally in terms of their biological attributions, in grapes and winery by-products, these compounds play an important role in sensorial characteristics (color, aroma, flavor, bitterness, or astringency), fostering new interests in health-promotion as a source of ingredients and additives or technological adjuvants [68], and thus are of great relevance to researchers, producers, processors, and consumers [69].

The various groups of phenolic compounds described so far in winery by-products are not uniformly distributed in the plant material, being distributed within subcellular compartments [70,71] as part of the different metabolic pathways involved in the plant cell defense to stress and pathogens [66]. In addition, phenolic compounds obtained from wine-industry residues belong to different classes, including phenolic acids (hydroxybenzoic acids and hydroxycinnamic acids), flavonoids (flavanols or flavan-3-ols, proanthocyanidins, flavones, and flavonols), and stilbenes [72], which have been reported as responsible for multiple biological effects [73].

The data available on total phenolic contents in vinification residues have been reported by analyzing wastes from different varieties grown under distinct agro-climatic conditions. Grape pomace is one of the most extensively analyzed materials as a whole, as well as for its separate components, mainly skins and seeds [2,4,8,9,17,18,27,28,30,31,32,33,34]. Content in total phenolics in seed extracts assessed by colorimetric assays showed a wide range of variation between cultivars ranging from 88.11 to 667.98 mg·GAE·g^−1^·dw [74,75]. These variations have been attributed to the concomitant effect of a number of factors including the genetic potential for polyphenol biosynthesis, the maturation stage, and the agro-climatic conditions [76,77]. On the other hand, the analysis of the phenolic composition of grape skins has revealed very similar contents in total phenolics from different grape varieties of up to 112 mg·GAE (gallic acid equivalent)·g^−1^·dw [8,78]. Total phenolics contents have been also estimated in grape stems residues by using colorimetric methods that allow recording of concentrations ranging from 26.88 to 35.99 mg·GAE·g^−1^·dw, and variations from one cultivar to another [79]. Distinct extraction procedures, grape varieties, and agro-climatic conditions contributed to differences in the quantified total polyphenols, including tannins and polymeric proanthocyanidins, with increased contents in stems and at least partially explaining the differences between the total phenolics reported in literature [22].

### 3.1. Phenolic Acids

Phenolic acids include benzoic and cinnamic acid derivatives, with hydroxycinnamic acids being the most abundant class in wine industry by-products. These compounds frequently appear in a conjugated form, namely as glycosylated derivatives or esters of quinic acid, shikimic acid, and tartaric acid [72]. To date, the relative proportion of the phenolic acids in winery by-products compounds have been calculated by HPLC analyses, existing data on residues from red and white cultivars that evidence significant differences between grape types (Table 1 and Table 2) and the distinct fraction of residues, namely stems, skins, seeds, pomace, or leaves). The genotype appears to be the major factor influencing the relative concentrations of the different phenolic compounds [80].

#### 3.1.1. Hydroxybenzoic Acids

Hydroxybenzoic acid derivatives identified so far, in this industrial residue, are mainly represented by *p-*hydroxybenzoic acid, protocatechic acid, tannic acid, vanillic acid, gallic acid derivatives, and syringic acid (Table 1 and Table 2) [68,72]. In this regard, gallic acid is described as the most abundant hydroxybenzoic acid derivative in grape stems, skins and, seeds, followed by syringic acid in grape stems, and protocatecuic acid in grape seeds and skins [65,81,82]. Gallic acid is especially relevant due to its role as a precursor of hydrolysable tannins [83]. Interestingly, the analysis of grape seeds and pomace from red varieties revealed the protocatechuic acid as the most abundant hydroxybenzoic acid (98.65 mg·g^−1^·dw, on average), with higher concentration than the other hydroxybenzoic acid derivatives described (Table 1). The differences concerning the phenolic profile in grape pomace and its individual constituents may be supported by the remaining grape pulp, as part of the grape pomace, which could provide additional phenolic content. Seeds from white varieties showed high contents in both gallic acid and protocatechuic acid with no significant differences between them [84].

The analysis of the individual hydroxybenzoic acids in the wine by-products considered in this review (Table 1 and Table 2) provides established differences between various residues concerning the agro-climatic conditions linked to their primary production. Thus, Singleton *et al*. (1978), described protocatechic acid as the only hydroxybenzoic derivative acid present in grapes skins, presenting the *trans-*isomers at higher concentrations than the *cis-*isomers [85].

#### 3.1.2. Hydroxycinnamic Acids

Hydroxycinnamic acids are found in all parts of grape fruit, with the highest recorded content being in the external tissues of the ripe fruit (grape skins). The concentration in hydroxycinnamic acids generally decrease during the ripening process, however, the total amount of this phenolic class increases proportionally to the fruit size.

The main hydroxycinnamic acids found in grapes and wines are caftaric, *p*-coutaric, and fertaric acids. Caftaric and fertaric acids are mainly found in the *trans-*form, while a negligible fraction of *p*-coutaric acid has been found in the *cis-*form. This profile in phenolic acids of organic material derived from the vinification process showed a different composition depending on the specific waste considered and the grape type (red or white) (Table 1 and Table 2). Grape stems from both red and white varieties showed *trans*-caftaric acid as the most abundant compound in this class [65,81]. The *trans*-isomers presented higher concentrations than the *cis*-ones in all cases. In this regard, Singleton *et al*. (1978) assumed that the *trans*-configuration of phenolic acids is the natural configuration, whereas the *cis*-form is the product of UV-induced isomerization [85].

Concerning to grape skins, the data available on the hydroxycinnamic acids in red and white varieties suggested the existence of critical differences. Whereas skins from white grapes are dominated by the content in *cis*-coutaric acid and *trans*-caftaric acid [82], this residue from red grapes, in addition to displaying much lower concentration of this type of phenolics, is mainly represented by chlorogenic acid [32]. In this way, no hydroxycinnamic acids have been described in seeds from white varieties whereas in those from red ones they were represented by chlorogenic acids in a relevant concentration, ranging from 2.90 to 6.80 mg·g^−1^·dw, [33]. In addition, the analyses of the grape pressing residues (grape pomace, which involved both skins and seeds, and a limited amount of grape stems and pulp) could provide interesting information on the phenolic composition of this material to support further rational valorization processes, however, to the best of our knowledge no descriptions of this phenolic class has been published on these vinification residues.

Finally, concerning grapevine leaves, which some authors do not consider as a residue even though they constitute an indubitable plant material produced as consequence of the wine agro-industrial activity, again, it is interesting to highlight the contents in *trans*-caftaric acids in both red and white varieties [59,86], except in the varieties “Moscatel de Grano Menudo”, “Cencibel”, and “Shiraz”, in which the main acid is *trans*-coutaric [87].

### 3.2. Flavonoids

Flavonoids are low molecular weight compounds structurally composed by 15 carbon atoms [88]. So far, the presence of flavanols or flavan-3-ols, proanthocyanidins, flavones, flavanols, and anthocyanins has been described in industrial residues from grapes [68]. The separate classes of flavonoids differ in the oxidation degree of the central pyran-ring [89]. A significant biosynthesis of flavonols takes place during grape development and ripening. The greatest concentration of flavonols in grapes has been described 3–4 weeks post-veraison [90]. This physiological process, together with the harvest timing strongly contributes to the final qualitative and quantitative content in bioactive flavonoids in vinification residues and, therefore, the rational valorization procedures.

The comparison of the flavonoids composition among the residues of wine making showed that flavan-3-ols are in similar concentrations in skins and in seeds, whereas anthocyanins are mainly found in the skins [91].

**Table 1:** Content in phenolic acids (analytical technique employed) in winery residues from different red varieties of *Vitis vinifera* L.

Compound	Stem	Skin	Seed	Pomace	Leaves	Lees
**Hydroxybenzoic acid**						
Gallic acid	1.30–2.40 (µg·100 g^−1^·dw; HPLC–DAD) [92]; 0.07–33.00 (mg·g^−1^·dw, HPLC–DAD) [65,81,82]	–	6.80–9.80 (mg·kg^−1^·fw, HPLC–FD) [84]	≤3.97 (mg·g^−1^·dw, HPLC–UV) [17]; 0.05–0.19 (mg·g^−1^·dw, HPLC–DAD) [32]	–	–
*p*-Hydroxybenzoic acid	–	–	–	≤6.59 (mg·g^−1^·dw, HPLC–UV) [17]	–	–
Protocatecuic acid	–	1.50–2.40 (mg·kg^−1^·fw, HPLC–FD) [83]	3.30–8.70 (mg·kg^−1^·fw, HPLC–FD) [84]	≤98.65 (mg·g^−1^·dw, HPLC–UV) [17]	–	–
Syringic acid	≤32.20 (mg·g^−1^·dw, HPLC–DAD) [81]	–	–	–	–	–
Tannic acid	–	–	–	≤3.85 (mg·g^−1^·dw, HPLC–UV) [17]	–	–
Vanillic acid	–	–	–	≤0.59 (mg·g^−1^·dw, HPLC–UV) [17]	–	–
**Hydroxycinnamic acid**						
Caffeic acid	≤0.60 (mg·g^−1^·dw, HPLC–DAD) [81]; 0.60–1.90 (µg·g^−1^·dw; HPLC–DAD) [92]	–	–	–	–	663 (µg·g^−1^·dw, HPLC–DAD) [61]
Caftaric acid	–	–	–	–	≤7.32 (mg·g^−1^·fw, HPLC–UV) [86]	
*cis*-Caftaric acid	–	0.20–0.50 (mg·kg^−1^·fw, HPLC–FD) [83]	–	–	–	
*trans*-Caftaric acid	0.04–16.10 (mg·g^−1^·dw, HPLC–DAD) [65,81]; ≤40.00 (mg·kg^−1^·fw; HPLC–DAD) [44]	4.90–9.50 (mg·kg^-1^·fw, HPLC–FD) [83]	–	–	1.40–3.28 (mg·g^−1^·dw, HPLC–DAD) [59]	
Chlorogenic acid	–	≤0.23 (mg·g^−1^·dw, HPLC–DAD) [32]	2.90–6.80 (mg·g^−1^·dw, HPLC–DAD) [33]	–	–	
*p*-Coumaric acid	0.04–0.90 (mg·g^−1^·dw, HPLC–DAD) [81]; 0.90–2.20 (µg·g^−1^·dw, HPLC–DAD) [92]	–	–	–	–	2449 (µg·g^−1^·dw, HPLC–DAD) [61]
*trans*-Coumaroyltartaric acid	–	–	–	–	0.49–1.49 (mg·g^−1^·dw, HPLC–DAD) [59]	
Coutaric acid	≤4.50 (mg·kg^−1^·fw; HPLC–DAD) [44]	–	–	–	–	
*cis*-Coutaric acid	–	0.90–2.70 (mg·kg^−1^·fw, HPLC–FD) [83]	–	–	–	
*trans*-Coutaric acid	–	3.20–10.00 (mg·kg^−1^·fw, HPLC–FD) [83]	–	–	–	
Ferulic acid	≤2.50 (mg·g^−1^·dw, HPLC–DAD) [80]	–	–	–	–	

HPLC–DAD: High Performance Liquid Chromatography–Diode Array Detector; HPLC–FD: High Performance Liquid Chromatography–Fluorescence Detector; HPLC–UV: High Performance Liquid Chromatography–Ultraviolet Detector; dw, dry weight; fw, fresh weight.

**Table 2:** Content in phenolic acids (analytical technique employed) in winery residues from different white varieties of *Vitis vinifera* L.

Compound	Stem	Skin	Seed	Pomace	Leaves
**Hydroxybenzoic acid**					
Gallic acid	0.01–0.03 (mg·g^−1^·dw, HPLC–DAD) [92]; 1.05–22.60 (µg·g^−1^·dw; HPLC–DAD) [65,81]	≤1.20 (mg·g^−1^·dw, HPLC–UV) [81]	≤1.90 (mg·g^−1^·dw, HPLC–UV) [82]; 15.70–19.70 (mg·g^−1^·dw, HPLC–UV) [93]	<1.98 (mg·g^−1^·dw, HPLC–DAD) [94]	–
Gallic acid hexose	–	–	≤0.80 (mg·g^−1^·dw, HPLC–UV) [82]	–	–
Gallic acid dihexose	–	≤1.10 (mg·g^−1^·dw, HPLC–UV) [81]	≤1.20 (mg·g^−1^·dw, HPLC–UV) [82]	–	–
Protocatecuic acid	–	–	≤6.00 (mg·kg^−1^·fw, HPLC–FD) [84]; <1.10 (mg·g^−1^·dw, HPLC–UV) [49]		–
Syringic acid	≤0.10 (µg·g^−1^·dw, HPLC–DAD) [81]	–	–	–	–
**Hydroxycinnamic acid**						
Caffeic acid	≤0.05 (µg·g^−1^·dw, HPLC–DAD) [81]; 1.00–1.50 (mg·g^−1^·dw, HPLC–DAD) [92]	–	–	–	–
*cis*-Caftaric acid	–	≤1.30 (mg·kg^−1^·fw, HPLC–FD) [84]; ≤2.50 (mg·g^−1^·dw, HPLC–UV) [82]	–	–	–
*trans*-Caftaric acid	0.05–12.20 (mg·g^−1^·dw, HPLC–DAD) [65,81]	2.40–31.00 (mg·kg^−1^·fw, HPLC–FD) [84]; ≤4.00 (mg·g^−1^·dw, HPLC–UV) [82]	–	–	18.81–83.36 (mg·g^−1^·dw, HPLC–DAD) [59]
*p*-Coumaric acid	0.01–0.08 (µg·g^−1^·dw, HPLC–DAD) [81]; 0.02–0.03 (mg·g^−1^·dw, HPLC–DAD) [92]	≤2.0 (mg·g^−1^·dw, HPLC–UV) [82]	–	0.03 (mg·g^−1^·dw, HPLC–DAD) [94]	–
*trans*-Coumaroyltartaric acid	–	–	–	–	2.12–10.71 (mg·g^−1^·dw, HPLC–DAD) [59]
Coutaric acid-*O*-hexoside	–	2.27 (mg·g^−1^·dw, HPLC–UV) [82]	–	–	–
*cis*-Coutaric acid	–	1.60–5.90 (mg·kg^−1^·fw , HPLC–FD) [84]; ≤6.20 (mg·g^−1^·dw, HPLC–UV) [82]	–	–	–
*trans*-Coutaric acid	–	1.90–18.00 (mg·kg^−1^·fw, HPLC–FD) [84]; ≤0.30 (mg·g^−1^·dw, HPLC–UV) [82]	–	–	–
*trans*-Fertaric acid	–	≤2.60 (mg·kg^−1^·fw, HPLC–FD) [84]; ≤1.70 (mg·g^−1^·dw, HPLC–UV) [82]	–	–	–
*trans*-Ferulic acid	–	–	–	0.04 (mg·g^−1^·dw, HPLC–DAD) [94]	–

HPLC–DAD: High Performance Liquid Chromatography–Diode Array Detector; HPLC–FD: High Performance Liquid Chromatography–Fluorescence Detector; HPLC–UV: High Performance Liquid Chromatography–Ultraviolet Detector; dw, dry weight; fw, fresh weight.

#### 3.2.1. Flavonols

Flavonols are flavonoids characterized by the presence of a double bond between carbons C2 and C3, and a hydroxyl group in C3 [95]. Different sugars can be bound to the flavonols, producing glycosides, glucuronides, galactosides, and diglycosides (glucosylarabinoside, glucosylgalactoside, glucosylxyloside and glucosylrhamnoside) [96]. Flavonols can react with anthocyanins to produce more stable pigments, and with phenolic acids to participate in copigmentation phenomena [7,84].

The distribution of flavonols in the separate materials of vinification wastes (stems, seeds, skins, pomace, and leaves), according to the bibliography, show differences concerning the individual flavonols as well as to their relative proportions (Table 3 and Table 4) and, in general, those materials from red varieties present higher amounts of these compounds than white variety byproducts.

When analyzing the concentration of individual flavonols in red grape stems, it has been observed that a wide variety of flavonols, with remarkable contents of quercetin derivatives, are mainly represented by quercetin-3-*O*-glucuronide (up to 128 mg·g^−1^·dw) [8,45]. Other major flavonols described in grape stems are quercetin-3-*O*-glucoside, quercetin-3-*O*-galactoside, and quercetin-3-*O*-rutinoside [8,81]. The stems from red and white varieties showed a similar profile of flavonols, but the plant material from white varieties present a much lower concentration of compounds. The phytochemical profile of grape pomace flavonols is quite simple, with mainly quercetin-3-*O*-glucuronide being the predominant compound [97,98,99] (Table 3). The flavonol profile of the constituents of grape pomace as a whole (seeds, skins and some pulp) provides evidenceof higher amounts of phenolics than when considering the separate materials (mainly skins and seeds). Actually, in grape skins, the preponderance of quercetin-3-*O*-glucuronide (39.5 and 37.5 mg·kg^−1^·fw (fresh weight) in white and red grape skins, respectively) and quercetin-3-*O*-glucoside (44.0 and 43.0 mg·kg^−1^·fw, on average, in white and red grape skins, respectively), remain at similar high levels [100], and interestingly, to the best of our knowledge, no quantitative analysis of quercetin-3-*O*-galactoside has been performed on grape seeds from red varieties whereas seeds from white cultivars reported contents of this compound. The lack of data on the grape seeds content in flavonols support the idea of an insignificant contribution to their concentration in pomace in addition to the amounts in pulp or stems.

The presence of the flavanonols engeletin (C_21_H_22_O_10_), as well as the flavonols myricetin (C_15_H_10_O_8_) and laricitrin (C_16_H_12_O_8_) has been not detected in organic residues from white varieties. This could be due to the absence of the enzyme flavonoid-3',5'-hydroxylase in white grape varieties [90,96]. Concerning “3-*O*-glycosides” flavonol derivatives in red grapes, they include three different complete series according to the nature of the sugar moiety linked to the C3 position. The “3-*O*-glucosides” are the main derivative of the six flavonol aglycones (kaempferol, quercetin, isorhamnetin, myricetin, laricitrin, and syringetin), whereas the correspondent “3-*O*-galactoside” derivatives are found to be minor compounds. The “3-*O*-glucuronides” have been described as the third kind of red grapes’ flavonol derivatives, and normally account as minor compounds for all of the flavonol aglycones, with the exception of quercetin-3-*O*-glucuronide, which is as abundant as quercetin 3-*O*-glucoside [86,101].

**Table 3:** Content in flavan-3-ols and tannins, flavones, and flavonols in winery residues of red varieties of *Vitis vinifera* L.

Compound	Stem	Skin	Seed	Pomace	Leaves	Lees
**Flavan-3-ols and tannins**					
Catechin ^Z^	0.71–85.80 (mg·g^−1^·dw, HPLC–DAD ^Y^) [65,81,102] 1.24–2.58 (µg·g^−1^·dw; HPLC–DAD) [92] ≤60.00 (mg·kg^−1^·fw, HPLC–DAD) [44] 0.12–1.27 (mg·g^−1^·dw, HPLC–UV) [103]	8.50–25.0 (mg·kg^−1^·fw, HPLC–FD) [84] ≤13.20 (mg·g^−1^·dw, HPLC–DAD) [32] 0.01–10.00 (mg·g^−1^·dw, HPLC–FD) [104]	0.08–4.50 (mg·g^−1^·fw, HPLC–FD) [85,105] 0.27–1.17 (mg·g^−1^·dw, HPLC–DAD) [33]	1.12–1.50 (mg·g^−1^·dw, HPLC–DAD) [97]	71.00 (mg·kg^−1^·fw, HPLC–UV) [86]	–
Epicatechin	≤1.00–13.30 (mg·g^−1^·dw, HPLC–DAD) [44,61,78,106] ≤0.11 (mg·g^−1^·dw, HPLC–UV) [103]	6.20–13.00 (mg·kg^−1^·fw, HPLC–FD) [84] ≤1.10 (mg·g^−1^·dw, HPLC–FD) [104]	0.06–0.21 (mg·g^−1^·fw, HPLC–FD) [85,105] ≤0.47 (mg·g^−1^·dw, HPLC–DAD) [33]	≤2.01 (mg·g^−1^·dw, HPLC–UV) [17]	15.00 (mg·kg^−1^·fw, HPLC–UV) [86]	–
Epicatechin gallate	0.06–7.80 (mg·g^−1^·dw, HPLC–DAD) [65,81] 15.50–19.80 (mg·kg^−1^·fw, HPLC–FD) [44]	–	13.00–70.00 (mg·kg^−1^·fw, HPLC–FD) [85]	–	15.00 (mg·kg^−1^·fw, HPLC–UV) [86]	–
Epigallocatechin	1.50–5.40 (mg·kg^−1^·fw, HPLC–DAD) [44]	–	–	–	–	–
Procyanidin B1	6.20–13.73 (mg·g^−1^·dw, HPLC–DAD) [102] 0.25–1.96 (mg·g^−1^·dw, HPLC–UV) [103]	8.40–22.00 (mg·kg^−1^·fw, HPLC–FD) [82]	74.00–170.00 (mg·kg^−1^·fw, HPLC–FD) [85]	–	–	–
Procyanidin B2	0.11–5.10 (mg·g^−1^·dw, HPLC–DAD) [65,81] ≤0.09 (mg·g^−1^·dw, HPLC–UV) [103]	0.70–2.20 (mg·kg^−1^·fw, HPLC–FD) [82]	21.00–41.00 (mg·kg^−1^·fw, HPLC–FD) [85]	–	–	–
Procyanidin B3	0.14–20.50 (mg·g^−1^·dw, HPLC–DAD) [65,81] 0.04–0.23 (mg·g^−1^·dw, HPLC–UV) [103]	16.00–39.00 (mg·kg^−1^·fw, HPLC–FD) [84]	43.00–64.00 (mg·kg^−1^·fw , HPLC–FD) [85]	–	–	–
Procyanidin B4	–	–	33.00–80.00 (mg·kg^−1^·fw, HPLC–FD) [85]	–	–	–
Procyanidin trimmer C1	–	≤0.04 (mg·g^−1^·dw, HPLC–FD) [104]	0.20–0.30 (mg·g^−1^·dw, HPLC–FD) [105]	–	–	–
**Flavones**						
Luteolin	0.02–0.04 (µg·g^−1^·dw; HPLC–DAD) [92]	–	–	–	–	–
**Flavonols**						
Engeletin	Traces (HPLC–DAD) [44]	–	–	–	–	–
Isorhamnetin	–	–	–	≤ 0.20 (mg·g^−1^·dw, HPLC–DAD) [97]	–	–
Isrhm-3-*O*-Glc	–	11.00–48.00 (mg·kg^−1^·fw, HPLC–FD) [84]	–	0.06 (mg·g^−1^·dw, HPLC–DAD) [96] ≤0.10 (mg·g^−1^·dw, HPLC–DAD) [97]	–	–
Isrhm-3-*O*-Gluc	–	–	–	≤ 0.90(mg·g^−1^·dw, HPLC–DAD) [97]	–	–
Kaempferol	≤1.80 (mg·g^−1^·dw, HPLC–DAD) [81]	–	–	<0.01 (mg·g^−1^·dw, HPLC–DAD) [98] ≤0.01 (mg·g^−1^·dw, HPLC–DAD) [97]	–	–
K-3-*O*-Glc	Traces (HPLC–DAD) [44]	8.00–14.00 (mg·kg^−1^·fw, HPLC–FD) [84]	–	0.01 (mg·g^−1^·dw, HPLC–DAD) [98] ≤0.20 (mg·g^−1^·dw, HPLC–DAD) [97]	≤30.0 (mg·kg^−1^·fw, HPLC–UV) [86] 2.03–5.51 (mg·g^−1^·dw, HPLC–DAD) [59]	–
K-3-*O*-Gluc	Traces (HPLC–DAD) [44]	–	–	–	–	–
Laricitin	–	–	–	≤ 0.20 (mg·g^−1^·dw, HPLC–DAD)[97]	–	–
Myricetin	–	–	–	–	–	4292 (µg·g^−1^·dw, HPLC–DAD) [61]
Myr-3-*O*-Glc	Traces (HPLC–DAD) [44]	13.00–26.00 (mg·kg^−1^·fw, HPLC–FD) [84]	–		0.49–1.49(mg·g^−1^·dw, HPLC–DAD) [59]	–
Myr-3-*O*-Gluc	Traces (HPLC–DAD) [44]	5.80–10.00 (mg·kg^−1^·fw, HPLC–FD) [84]	–	–	–	–
Quercetin	0.60–8.20 (µg·g^−1^·dw, HPLC–DAD) [81]	–	–	15.30 (mg·kg^−1^·dw, HPLC–DAD)[97] 0.50–2.80 (mg·100 g^−1^·dw, HPLC–DAD) [97]	47.00 (mg·kg^−1^·fw, HPLC–UV) [86]	13,656 (µg·g^−1^·dw, HPLC–DAD) [61]
Q-3-*O*-Gal	6.60–15.00 (mg·g^−1^·dw, HPLC–DAD) [81]	–	–	–	–	–
Q-3-*O*-Glc	0.28–7.30 (mg·g^−1^·dw, HPLC–DAD) [81,102] 18.0 (mg·kg^−1^·fw, HPLC–DAD) [44]	31.00–55.00 (mg·kg^−1^·fw, HPLC–FD) [84]	–	0.18 (mg·g^−1^·dw, HPLC–DAD) [98] ≤0.90–5.11 (mg·g^−1^·dw, HPLC–DAD) [97,99]	142.00 (mg·kg^−1^·fw, HPLC–UV) [86]	–
Q-3-*O*-GlcXyl	–	9.00–18.00 (mg·kg^−1^·fw, HPLC–FD) [84]	–	–	–	–
Q-3-*O*-Gluc	0.20–126.80 mg·g^−1^·dw, HPLC–DAD) [44,102]	29.00–59.00 (mg·kg^−1^·fw, HPLC–FD) [84]	–	≤1.30–130.00 (mg·g^−1^·dw, HPLC–DAD) [97,98] ≤105.40 (mg·g^−1^·dw, HPLC–DAD) [99]	–	8900 (µg·g^−1^·dw, HPLC–DAD) [61]
Q-3-*O*-Rha	0.30–2.80 (mg·g^−1^·dw, HPLC–DAD) [81]	–	–	–	–	–
Q-3-*O*-Rut	1.90–41.80 (mg·g^−1^·dw, HPLC–DAD) [81]	0.01–0.57 (mg·g^−1^·dw, HPLC–DAD) [32,99]	–	0.12–0.50 (mg·g^−1^·dw, HPLC–DAD) [33,97]	–	–
**Flavanonols**					
Astilbin	35.00(HPLC–DAD; mg·kg^−1^·fw) [44]	–	–	–	–	–

^Z^ Gal: Galactoside, Glc: glucoside, Gluc: Glucuronide, Isrhm: Isorhamntin, K: Kaempferol, Myr: Myricetin, Q: Quercetin, Rha: Rhamnoside, Rut: Rutinoside, Xyl: Xyloside; ^Y^ HPLC–DAD: High Performance Liquid Chromatography–Diode Array Detector, HPLC–FD: High Performance Liquid Chromatography–Fluorescence Detector, HPLC–UV: High Performance Liquid Chromatography–Ultraviolet Detector; dw, dry weight; fw, fresh weight.

**Table 4:** Content in flavan-3-ols and tannins, flavones, and flavonols in winery residues of white varieties of *Vitis vinifera* L.

Compound	Stem	Skin	Seed	Pomace	Leaves
**Flavan-3-ols and tannins**						
Catechin ^Z^	46.50–98.30 (µg·g^−1^·dw, HPLC–DAD ^Y^) [84] 0.13–2.89 (mg·g^−1^·dw, HPLC–DAD) [92,93] 3.85–18.58 (µg·g^−1^·dw; HPLC–DAD) [65] 9.30–133.90 (mg·g^−1^·dw, HPLC–UV) [103]	≤23.00 (mg·kg^−1^·fw, HPLC–FD) [84] 11.40 (mg·g^−1^·dw, HPLC–UV) [82] 0.72–0.84 (mg·g^−1^·dw, HPLC–DAD) [93]	120.00–500.00 (mg·kg^−1^·fw, HPLC–FD) [84] 106.50 (mg·g^−1^·dw, HPLC–UV) [82] 83.1–98.3 (mg·g^−1^·dw, HPLC–UV) [107] 0.45–0.53 (mg·g^−1^·dw, HPLC–DAD) [93]	<0.30 (mg·g^−1^·dw, HPLC–DAD) [108,109]	–
Dimer gallate	–	–	38.1–46.8 (mg·g^-1^·dw, HPLC–UV) [107]	–	–
Epicatechin	≤4.00–0.58 (µg·g^−1^·dw, HPLC–DAD) [65,81] 0.04–1.13 (mg·g^−1^·dw, HPLC–DAD) [93,110] 0.50–5.80 (mg·g^−1^·dw, HPLC–UV) [103]	≤8.30 (mg·kg^−1^·fw, HPLC–FD) [84] 2.70 (mg·g^−1^·dw, HPLC–UV) [82] 0.77–0.85 (mg·g^−1^·dw, HPLC–DAD) [93]	110.00–310.00 (mg·kg^−1^·fw, HPLC–FD) [84] 77.50 (mg·g^−1^·dw, HPLC–UV) [82] 54.2–55.8 (mg·g^−1^·dw, HPLC–UV) [107] 0.79–0.89 (mg·g^−1^·dw, HPLC–DAD) [107]	<0.07 (mg·g^−1^·dw, HPLC–DAD) [108]	–
Epicatechin-epicatechingallate I	–	–	26.80 (mg·g^−1^·dw, HPLC–UV) [82]	–	–
Epicatechin-epicatechingallate II	–	–	23.70 (mg·g^−1^·dw, HPLC–UV) [82]	–	–
Epicatechin-epicatechingallate III	–	–	21.40 (mg·g^−1^·dw, HPLC–UV) [82]	–	–
Epicatechin gallate	0.34–15.70 (µg·g^−1^·dw, HPLC–DAD) [44,65,81]	–	13.00–67.00 (mg·kg^−1^·fw, HPLC–FD) [84] 76.50 (mg·g^−1^·dw, HPLC–UV) [82] 53.8–56.8 (mg·g^−1^·dw, HPLC–UV) [107]	<0.05 (mg·g^−1^·dw, HPLC–DAD) [108]	–
Epicatechin-3-hexose	–	–	1.50 (mg·g^−1^·dw, HPLC–UV) [82]	–	–
Epigallocatechin	0.80–0.90 (mg·kg^−1^·fw, HPLC–DAD) [44]	2.10 (mg·g^−1^·dw, HPLC–UV) [82]	–	–	–
Epigallocatechin gallate	–	–	0.30–0.50 (mg·g^−1^·dw, HPLC–UV) [107]	–	–
Epigallocatechin-epicatechin	–	–	3.60 (mg·g^−1^·dw, HPLC–UV) [82]	–	–
Procyanidin dimmer B1	13.30–187.70 (mg·g^−1^·dw, HPLC–UV) [103] 0.07–0.11 (mg·g^−1^·dw, HPLC–DAD) [93]	≤48.00 (mg·kg^−1^·fw, HPLC–FD) [84] 0.36–0.48 (mg·g^−1^·dw, HPLC–DAD) [93]	200.00–620.00 (mg·kg^−1^·fw, HPLC–FD) [82] 3.10 (mg·g^−1^·dw, HPLC–UV) [82] 0.10–0.14 (mg·g^−1^·dw, HPLC–DAD) [107]	<0.22 (mg·g^−1^·dw, HPLC–DAD) [93,108]	–
Procyanidin dimmer B2	0.02–8.50 (mg·g^−1^·dw, HPLC–DAD) [65,81,93] 1.10–4.80 (mg·g^−1^·dw, HPLC–UV) [103]	8.70 (mg·g^−1^·dw, HPLC–UV) [82] 0.59–0.65 (mg·g^−1^·dw, HPLC–DAD) [93]	15.00–33.00 (mg·kg^−1^·fw, HPLC–FD) [84] 64.50 (mg·g^−1^·dw, HPLC–UV) [82] 0.79–0.91 (mg·g^−1^·dw, HPLC–DAD) [107]	<0.06 (mg·g^−1^·dw, HPLC–DAD) [93,108]	–
Procyanidin dimmer B3	0.02–31.60 (mg·g^−1^·dw, HPLC–DAD) [65,81,93] 4.50–22.20 mg·g^−1^·dw (HPLC–UV) [111]	≤37.00(mg·kg^−1^·fw, HPLC–FD) [84] 0.31–0.35 (mg·g^−1^·dw, HPLC–DAD) [93]	39.00–56.00 (mg·kg^−1^·fw, HPLC–FD) [84] 44.60 (mg·g^−1^·dw, HPLC–UV) [82] 0.11–0.15 (mg·g^−1^·dw, HPLC–DAD) [107]	<0.06 (mg·g^−1^·dw, HPLC–DAD) [108]	–
Procyanidin dimmer B4	0.03–0.05 (mg·g^−1^·dw, HPLC–DAD) [93]	8.00 (mg·g^−1^·dw, HPLC–UV) [82] 0.26–0.30 (mg·g^−1^·dw, HPLC–DAD) [93]	40.00–95.00 (mg·kg^−1^·fw, HPLC–FD) [84] 58.40 (mg·g^−1^·dw, HPLC–UV) [82] 0.21–0.31 (mg·g^−1^·dw, HPLC–DAD) [107]	<0.07 (mg g^−1^ dw, HPLC–DAD) [108]	–
Procyanidin B1-*O*-gallate	0.04 (mg·g^−1^·dw, HPLC–DAD) [93]	0.31–0.35 (mg·g^−1^·dw, HPLC–DAD) [93]	0.66–0.82 (mg·g^−1^·dw, HPLC–DAD) [107]	–	–
Procyanidin B2-*O*-gallate	0.02 (mg·g^−1^·dw, HPLC–DAD) [93]	0.27–0.30 (mg·g^−1^·dw, HPLC–DAD) [93]	0.66–0.79 (mg·g^−1^·dw, HPLC–DAD) [107]	<0.14 (mg·g^−1^·dw, HPLC–DAD) [108]	–
Procyanidin trimmer C1	0.11–0.19 (mg·g^−1^·dw, HPLC–DAD) [93]	0.35 (mg·g^−1^·dw, HPLC–DAD) [93]	12.70–31.40 (mg·g^−1^·dw, HPLC–UV) [82] 0.51–0.61 (mg·g^−1^·dw, HPLC–DAD) [107]	<0.03 (mg·g^−1^·dw, HPLC–DAD) [108]	–
Procyanidin trimmer C2	–	–	18.50 (mg·g^−1^·dw, HPLC–UV) [82]	<0.04 (mg·g^−1^·dw, HPLC–DAD) [108]	–
Procyanidin trimmer C3	–	–	13.50 (mg·g^−1^·dw, HPLC–UV) [82]	–	–
Galloyled-procyanidin	–	–	–	<0.07 (mg·g^−1^·dw, HPLC–DAD) [108]	–
Gallocatechin-catechin dimer	–	–	–	<0.03 (mg·g^−1^·dw, HPLC–DAD) [108]	–
Procyanidin tetramer	–	–	1.80–2.20 (mg·g^−1^·dw, HPLC–UV) [107]	<0.06 (mg·g^−1^·dw, HPLC–DAD) [108]	
**Flavones**						
Luteolin	0.01–0.07(µg·g^−1^·dw, HPLC–DAD) [16]	–	–	–	–
**Flavonols**						
Isrhm-3-*O*-Glc	–	≤2.30 (mg·kg^-1^·fw, HPLC–FD) [84]	–	<0.01 (mg·g^−1^·dw, HPLC–DAD) [108]	–
Kaempferol	0.04–0.20 (µg·g^−1^·dw, HPLC–DAD) [81]	3.2 (mg·g^−1^·dw, HPLC–UV) [82]	–	–	–
K-3-*O*-Glc	–	2.00–26.00 (mg·kg^−1^·fw, HPLC–FD) [84] 8.40 (mg·g^−1^·dw, HPLC–UV) [82] 0.10 (mg·g^−1^·dw, HPLC–UV) [107]	–	<0.02 (mg·g^−1^·dw, HPLC–DAD) [108]	34.79–62.04 (mg·g^−1^·dw, HPLC–DAD) [59]
K-3-*O*-Gluc	3.20 (mg·g^−1^·dw, HPLC–UV) [82]	–	–	<0.01 (mg·g^−1^·dw, HPLC–DAD) [108]	–
Myr-3-*O*-Glc	–	–	–	–	1.11–8.50 (mg·g^−1^·dw, HPLC–DAD) [59]
Quercetin	0.30–2.20 (µg·g^−1^·dw, HPLC–DAD) [81]	0.30 (mg·g^−1^·dw, HPLC–UV) [107]	–	–	–
Q-3-*O*-Gal	13.50–19.20 (µg·g^−1^·dw, HPLC–DAD) [81]	–	0.20 (mg·g^−1^·dw, HPLC–UV) [107]	<0.02 (mg·g^−1^·dw, HPLC–DAD) [108]	–
Q-3-*O*-Glc	4.50–7.20 (µg·g^−1^·dw, HPLC–DAD) [81]	8.90–66.00 (mg·kg^−1^·fw, HPLC–FD) [84] 12.40 (mg·g^−1^·dw, HPLC–UV) [82]	–	<0.11 (mg·g^−1^·dw, HPLC–DAD) [108]	–
Q-3-*O*-Gluc	–	12.00–67.00 (mg·kg^−1^·fw, HPLC–FD) [84] 1.00 (mg·g^−1^·dw, HPLC–UV) [82]	–	<0.09 (mg·g^−1^·dw, HPLC–DAD) [108]	–
Q-3-*O*-pentoside	–	0.20 (mg·g^−1^·dw, HPLC–UV) [82]	–	–	–
Q-3-*O*-Rha	0.30–1.90 (µg·g^−1^·dw, HPLC–DAD) [81]	–	–	–	–
Q-3-*O*-Rut	–	0.40 (mg·g^−1^·dw, HPLC–UV) [7]	–	<0.02 (mg·g^−1^·dw, HPLC–DAD) [108]	–
**Flavanonols**						
Astilbin	–	5.60 (mg·g^−1^·dw, HPLC–UV) [80]	–	–	–

^Z^ Gal: Galactoside, Glc: glucoside, Gluc: Glucuronide, Isrhm: Isorhamntin, K: Kaempferol, Myr: Myricetin, Q: Quercetin, Rha: Rhamnoside, Rut: Rutinoside; ^Y^ HPLC–DAD: High Performance Liquid Chromatography–Diode Array Detector, HPLC–FD: High Performance Liquid Chromatography–Fluorescence Detector, HPLC–UV: High Performance Liquid Chromatography–Ultraviolet Detector; dw, dry weight; fw, fresh weight.

Grapevine leaves have been scarcely evaluated on their content in phenolics (Table 1, Table 2, Table 3, Table 4, Table 5, Table 6 and Table 7). Actually, the studies available on the flavonol composition of grapevine leaves highlighted the presence of glucoside derivatives in high concentrations, surpassing the content recorded in the other vinification residues (stems, skins, seeds, and pomace) [59,87]. The presence of flavonoids in leaves has been pointed out as a plant physiologic strategy related with their capacity to act as UV filters, protecting some cell structures, like chloroplasts, from harmful effects of UV radiation [112].

#### 3.2.2. Flavanols

Flavanols, also called flavan-3-ols, are flavonoid type compounds that have a hydroxyl group at the C_3_ position and no carbonyl group at the C_4_ position. These types of flavonoids have been described from the point of view of the physiological role in plants as attractants to pollinators, dyers, and contributors to the organoleptic properties of plant foods. In wineries, flavanols are the major compounds responsible for the astringency, bitterness, and structure of wines [84,113]. On the other hand, tannins are complex phenolic compounds of high molecular weight that can be divided in two distinct groups: condensed tannins and hydrolysable tannins [90]. Condensed tannins, also known as proanthocyanidins, are constituted by subunits of flavan-3-ols monomers and their structures vary depending on their constitutive subunits, the degree of polymerization, and the linkage position [91], whereas hydrolyzable tannins are complex (poly)phenolics that can be degraded into smaller units, mainly sugars and phenolic acids [68].

The evaluation of winery by-products on their content in bioactive compounds as support for further rational applications has allowed to establish the differential concentration of flavan-3-ols and tannins in the separate fractions of vinification by-products (stems, seeds, skins, pomace, and leaves; Table 3 and Table 4).

The comparison of concentrations of flavan-3-ols in different grape stems establish that catechin is the major compound in all winery residues from both red and white grapes, and the concentrations in grape stems are higher than those described in skins and seeds. In this regard, the highest concentration of catechins described in red grape stems (85.80 mg·g^−1^·dw, on average) exceed those recorded in skins and seeds by up to 85%, on average. Concerning white grapes, in which catechins are also the most abundant flavan-3-ols in all tissues, grape stems are the best source with concentrations higher than the maximal contents described in skins or leaves (92% and 21% lower, on average, respectively).

**Table 5:** Content in anthocyanins of winery residues from different red varieties of *Vitis vinifera* L.

Compound	Skin	Pomace	Lees
Cy-3-*O*-Glc ^Z^	≤0.04 (mg·g^−1^·dw, HPLC–DAD ^Y^) [99] ≤1.00 (mg·g^−1^·dw, HPLC–FD) [104]	<0.01 (mg·g^−1^·dw, HPLC–UV–DAD) [97,99] 0.01–1.79 (mg·g^−1^·dw, HPLC–DAD) [99]	–
Cy-3-*O*-(6'-*O*-acetyl)-Glc	–	<0.01 (mg·g^−1^·dw, HPLC–UV–DAD) [99]	–
Del-3-*O*-Glc	0.02–0.19 (mg·g^−1^·dw, HPLC–DAD) [99] ≤4.2 (mg·g^−1^·dw, HPLC–FD) [104]	≤1.20 (mg·g^−1^·dw, HPLC–UV–DAD) [97,99] 0.03–4.24 (mg·g^−1^·dw, HPLC–DAD) [99]	–
Del-3-*O*-(6'-*O*-*p*-coumaryl)-Glc	–	≤2.2 (mg·g^−1^·dw, HPLC–UV–DAD) [97]	–
Mv-3-*O*-Glc	12.10–16.50 (mg·g^−1^·dw, HPLC–DAD) [99]	0.06–10.40 (mg·g^−1^·dw, HPLC–UV–DAD) [97,99] 0.095–14.61 (mg·g^−1^·dw, HPLC–DAD) [99]	0.091 (mg·g^−1^·dw, HPLC–DAD) [61]
Mv-3-*O*-(6'-*O*-acetyl)-Glc	–	≤2.0 (mg·g^−1^·dw, HPLC–UV–DAD) [97,99]	–
Mv-3-*O*-(6'-*O*-caffeoyl)-Glc	29.0–59.0 (mg·kg^−1^·fw, HPLC–FD) [104]	≤0.3 (mg·g^−1^·dw, HPLC–UV–DAD) [97,99]	–
Mv-3-*O*-(6'-*p*-coumaroyl)-Glc	–	≤27.10 (mg·g^−1^·dw, HPLC–UV–DAD) [97,99] ≤0.10 (mg·g^−1^·dw, HPLC–DAD) [99]	11.7 (mg·g^−1^·dw, HPLC–DAD) [61]
Pn-3-*O*-Glc	1.90–7.10 (mg·g^−1^·dw, HPLC–DAD) [99] 0.30–11.50 (mg·g^−1^·dw, HPLC–FD) [104]	0.02 (mg·g^−1^·dw, HPLC–UV–DAD) [99] 0.10–1.50 (mg·g^−1^·dw, HPLC–UV–DAD) [97] 0.03–3.47 (mg·g^−1^·dw, HPLC–DAD) [99]	–
Pn-3-*O*-Gal	0.02–0.10 (mg·g^−1^·dw, HPLC–DAD) [99]	–	–
Pn-3-*O*-(6''-*O*-*p*-coumaryl)-Glc	–	≤1.20 (mg·g^−1^·dw, HPLC–UV–DAD) [97,99]	–

^Z^ Cy: Cyanidin, Del: Delphinidin, Gal: Galactoside, Glc: glucoside, Mv: Malvidin, Pn: Peonidin; ^Y^ HPLC–DAD: High Performance Liquid Chromatography–Diode Array Detector, HPLC–FD: High Performance Liquid Chromatography–Fluorescence Detector, HPLC–UV: High Performance Liquid Chromatography–Ultraviolet Detector; dw, dry weight; fw, fresh weight.

**Table 6:** Content in stilbenes (analytical technique employed) in winery residues of red grapes (*Vitis vinifera* L.).

Compound	Stem	Skin	Seed	Pomace	Leaf
*trans*-Res	≤0.09–124.10 (mg·g^−1^·dw, HPLC–DAD ^Y^) [65,81,92,102]	–	≤0.01 (mg·g^−1^·dw, HPLC–DAD) [33]	≤0.06 (mg·g^−1^·dw, HPLC–DAD) [33]	–
*trans*-Res-3-*O*-Glc ^Z^	–	–	–	–	≤96.00 (mg·kg^−1^·fw, HPLC–UV) [86]
*cis*-Res-3-*O*-Glc	–	–	–	–	≤129.00 (mg·kg^−1^·fw, HPLC–UV) [86]
ε-Viniferin	0.20–49.10 (mg·g^−1^·dw, HPLC–DAD) [65,81,102]	–	–	–	–

^Z^ Glc: glucoside, Res: Resveratrol; ^Y^ HPLC–DAD: High Performance Liquid Chromatography–Diode Array Detector; HPLC–UV: High Performance Liquid Chromatography–Ultraviolet detector; dw, dry weight; fw, fresh weight.

**Table 7:** Content in stilbenes (analytical technique employed) in winery residues of white grapes (*Vitis vinifera* L.).

Compound	Stem	Skin	Seed	Pomace	Leaf
*trans*-Piceid	–	≤6.90 (mg·g^−1^·dw, HPLC–UV) [82]	–	–	–
*trans*-Res ^Z^	≤0.02 (mg·g^−1^·dw, HPLC–DAD ^Y^) [65,81,92]	≤1.40 (mg·g^−1^·dw, HPLC–UV) [82]	–	–	–
ε-Viniferin	12.10–50.10 (mg·g^−1^·dw, HPLC–DAD) [81] 1.67–4.99 (µg·g^−1^·dw; HPLC–DAD) [65]	–	–	–	–

^Z^ Res: Resveratrol; ^Y^ HPLC–DAD: High Performance Liquid Chromatography–Diode array detector; HPLC–UV: High Performance Liquid Chromatography–Ultraviolet detector; dw, dry weight; fw, fresh weight.

Condensed tannins also represent a significant proportion of the bioactive phytochemicals in vinification residues and available records describe the presence of procyanidin dimers B1, B2, B3, and B4 and procyanidin trimers C1, C2, and C3. The analysis of the relative proportions as well as the absolute concentrations of these compounds in the winery by-products suggest critical differences in residues from red and white varieties which are of interest for the evaluation of the further uses of separate materials. Concerning red varieties, the highest content in condensed tannins is represented by the procyanidin dimer B3 (up to 20.50 mg·g^−1^·dw) [65,81], whereas they are almost absent in grape skins or seeds. However, data from white grape residues shows that although the highest content is found in stems (187.70 mg·g^−1^·dw), the concentrations are much higher than those described for red varieties, with procyanidin B1 as the major compound in white grapes. Thus, grape skins and seeds, also showed procyanidin dimer B2 as the most abundant compound with concentrations of total procyanidins up to 8.70 and 6.50 mg·g^−1^·dw, respectively (Table 4). Standardized grape seed extracts contain between 74% and 78% oligomeric proanthocyanidins, on a dry weight basis [113], which can combine with gallic acid to form gallate esters, and ultimately glycosides [8,114].

Based on these facts, the differences between the main procyanidin in the different grape tissues could seem contradictory. In order to understand these differences it is necessary to take into account that, in the case of procyanidins B3 and B4, certain deviations in the results reported is possible, since these were quantified with the response factor of procyanidin B1 given to the lack of specific commercial standards. This response factor in the HPLC methods varies for available standards of procyanidins B1 and B2 and, therefore, a similar variation would be likely for the response factor of procyanidins B3 and B4 [87].

Condensed tannins represent a quantitatively important value of the pomace composition (21%–52% of the dry weight matter) even though, until now, the number of publications on the quantification of individual compounds in grape pomace does not allow the comparison with other tissues. Proanthocyanidin oligomers are presumably linked through covalent bonding to cell wall polysaccharide materials, and may explain the high tannin content of the residual pomace [4].

Additional effects of the climate factors should be considered to explain the apparent discrepancy between separate studies. Regarding this matter, analyses of grape procyanidins from plant material obtained in regions with hot summers (typical of Southern Spain and Greece) [106,110], and less extreme summers (Northern Spain and Southern France) [115,116] suggest that climate conditions are also responsible for the differences of the procyanidin patterns.

#### 3.2.3. Flavones

Flavones are compounds with a double bond between carbon C2 and C3 that differ from flavonols by the absence of the hydroxyl group in carbon 3. These phytochemicals have been described in non-significant amounts in grapes with the exception of luteolin [68]. Hence, the presence of luteolin has also been reported in winery residues, however, the concentration reported by Çetin *et al*. (2007) in grape stems is in the range of “µg·g^−1^·dw” with no significant differences between red and white varieties in the hallmark of an evaluation work performed on 10 Turkish cultivars [92].

#### 3.2.4. Anthocyanins

Anthocyanins are the highly soluble flavonoids directly responsible for color in red grapes and wines, and for which the characteristics are determined by the chemical structure, existing mainly in the stable red coloured form (the flacylium cation form) at low pH values, degree of hydroxylation, methylation and glycosylation [68,84]. These compounds consist of the aglycone anthocyanidin (cyanidin, delphinidin, and peonidin, among others), an aromatic ring (A) bonded to an heterocyclic ring (C) that contains oxygen, which is also bonded by a carbon–carbon bond to a third aromatic ring (B). This aromatic ring B forms conjugates with sugars and organic acids to generate a multitude of anthocyanins of differing colors. Anthocyanins are relatively unstable and easily oxidized. They are sensitive to many factors, apart from pH, that may affect their stability and colour, such as temperature and UV radiation [117].

Grape skin is the specific part of grape with major content in these bioactive colored flavonoids, this tissue being the most responsible for the transferring of pigments to the wine [82]. Thus, the quantification of anthocyanidins in vinification by-products has been performed on grape skins and pomace (Table 5).

Available data on the distribution of these compounds in grape skin and pomace show that malvidin-3-*O*-glucoside is the main anthocyanin found with concentrations up to 16.50 and 10.40 mg·g^−1^·dw, respectively [98,99]. The second most abundant anthocyanin is peonidin-3-*O*-glucoside, which shows large variations from 0.70 to 11.50 mg·g^−1^·dw in these tissues [104]. Other minor anthocyanins listed in Table 5 have been recorded in much lower concentrations as widely reported [97,98,99,104]. However, a highly relevant event concerning coloured flavonoids is the biochemical transformation of these compounds during the elaboration of wine and even during wine storage. Hence, during the first steps of fermentation in the winemaking process, anthocyanin-derived pigments, basically pyranoanthocyanins (A- and B-type vitisins and hydroxyphenyl-pyranoanthocyanins derived from p-coumaric acid), are formed by the reaction of anthocyanidin-3-glucosinde with compounds of low molecular weight such as 4-vinylphenol, pyruvic acid, and flavonols [105,118]. However, these compounds have been detected in red wines and wine by-products in very minor concentrations, and may be absorbed only by the lees, which display a content of 1.2–2 g·kg^−1^·dw, and not detected in others grape tissues or vinification residues such as grape skin and seeds [119]. Pérez-Serradilla *et al*. (2011) indicate that antioxidant extracts from wine lees can be a suitable and cheap alternative to those obtained from grape seeds or skins [120].

### 3.3. Stilbenes

Stilbenes are phenolic compounds chemically constituted by two aromatic rings linked by an ethylene bridge [68]. It can be found in wine by-products, mostly in grape skin, but also in stems and seeds but in lower abundance [10,121]. To date, the presence of *trans*-piceid, *trans*-resveratrol, *trans*-resveratrol-3-*O*-glucoside, *cis*-resveratrol-3-*O*-glucoside, and ε-viniferin has been described.

Resveratrol is present in the different organic residues of vinification procedures; the wine processing methods determine its concentration in the final product [122,123,124]. On this matter, grapes produce stilbenes in response to a number of physiological stressing factors, including ozone and UV-C radiation, increasing stilbene levels up to several hundred-folds in grape skins and leaves [125]. Therefore, it may be possible to modify the stilbene contents of grape residues by initial industrial processes used to obtain the must.

The evaluation of the separate organic residues from the winery industry has shown that the content of stilbenes presents a different profile concerning red and white grapes. In this sense, whereas the presence of stilbene derivatives in stems, seeds, pomaces and leaves from red varieties, in white cultivars has been described, they have been detected only in stems and skins [33,65,81,82,86,92,102] (Table 6 and Table 7).

The absence of stilbenes in red grape skins as well as their presence only as *trans* isomers on white grape skins may be influenced by the performance of the extraction procedure (in darkness) since exposure of grapes to UV radiation transforms *trans*-isomers into *cis*-isomers [82].

The data available on the concentration of the separate stilbene derivatives indicates that, in red grapes, the highest content corresponded to *trans*-resveratrol and ε-viniferin in grape stems (up to 124.10 and 49.10 mg·g^−1^·dw, respectively) (Table 6), which largely surpasses the concentration recorded in seeds and pomace. Leaves from red varieties do not present these two bioactive stilbenes, showing the presence of the glycosylated forms in the range of 96–129 mg·kg^−1^·fw [86]. On the other hand, grape stems from vinification processes developed on white grapes present lower concentrations of both *trans*-resveratrol and ε-viniferin (Table 7).

In grapes, *trans*-resveratrol is distributed mostly in the skin [111], even though the concentration of *trans*-resveratrol in grape skins and seeds varies considerably within *Vitis* germplasm [126]. It is worth knowing all resveratrol derivatives in grape by-products in order to support rational valorization of vinification residues for their use in the future. However, only one or two derivatives have been studied along with other phenolic compounds [111,126,127].

## 4. Biological Activities and Potential Health Benefits of Winery Wastes Polyphenols

By-products derived from wine making process contain a high amount of secondary metabolites including phenolic acids, flavanols, proanthocyanidins, flavonols, anthocyanins, and stilbenes [46] (Table 1, Table 2, Table 3, Table 4, Table 5, Table 6 and Table 7). To date, there is a great deal of evidence concerning biological activities, such as antioxidant, antimicrobial, anti-inflammatory, anticancer, and cardiovascular protection activities, in support of phenolic compound use in pharmaceutical, food, and cosmetic industries [103,128].

One of the most cited biological activities attributed to the phenolic compounds is based on their antioxidant capacity, preventing the oxidation of LDL lipoproteins, platelet aggregation, and the damage of red blood cells through radical scavenging [129]. Additionally, phenolics are metal chelators, antimutagens/anticarcinogens, antimicrobials, and anti-inflammatory agents [27,46,130].

The radical scavenging activities of phenolic compounds are supported by their reducing power through hydrogen donating and single oxygen quenching. These activities allow these compounds to interact with biological systems, preventing degenerative diseases linked to oxidative stress in separate tissues and organic systems [10,104].

The antioxidant capacity of the (poly)phenolics in vinification residues, to act against oxidative damage [131], support the dietary intake of food products with high content in these natural antioxidants associated with lower prevalence/severity of pathophysiological conditions [132,133]. The capacity of the (poly)phenolics in grape derived products to prevent radical oxidation of the polyunsaturated fatty acids of low-density lipoproteins (LDL), which occurs primarily through oxidative modification of the apoprotein toward an atherogenic form, has a direct consequence on the prevention of cardiovascular diseases, appearing to play a major role in this degenerative process [134]. The link between the reduction of oxidized LDL particles and the presence of antioxidant phenolics has been demonstrated *in vitro* and *in vivo* and provides evidence for the reduced rates of cardiac diseases in populations consuming diets rich in antioxidant phenolics [135,136,137].

Anthocyanins contribute more to the antioxidant capacity of fruits (90%) than flavonols, flavan-3-ols, and phenolic acids (10%) [138]. On this matter, grape skins have been highlighted for their antioxidant and anti-glycation activities because of their anthocyanins and proanthocyanidins content [2,33]. Recent reports showed the antioxidant properties of grape seeds both *in vivo* and *in vitro* [21], which has been primarily attributed to flavonoids [139,140], and closely dependent on their chemical structure when present in plant material. Thus, whereas hydroxyl (–OH) groups enhance the activity, their substitution by carboxyl (–OCH_3_) groups diminishes the antioxidant potential [141]. Furthermore, the degree of polymerization of the procyanidins may also determine the antioxidant capacity [142]. The antioxidant capacity of selected grape stem extracts correlated well with the capability to prevent the oxidation of LDL lipoproteins at very low concentrations of (poly)polyphenolics. Thus, grape stem extracts have been suggested for their potential for reducing intracellular ROS levels. However, the poor correlation observed between (poly)phenolics composition and the ability of stem extracts to scavenge intracellular ROS may indicate that total phenolic content could be a poor indicator for the prediction of the antioxidant capacity at the cellular level, which is rationalized by the complexity of mechanisms that occur in living cells [143].

Some preclinical *in vivo* studies on the capacity of winery residues to prevent LDL oxidation has shown that a 15% grape pomace in a cholesterol diet (0.3%) is able to reduce the rat liver and serum levels of cholesterol by half, while increasing high-density liproprotyeins (HDL) by 26% [144]. Thus, grape seed extracts have been promoted as a source of (poly)phenols able to reduce the risk of heart disease by inhibiting the oxidation of LDL, improving endothelial function, lowering blood pressure, inhibition of platelet aggregation, reducing inflammation and activating novel proteins that prevent cell senescence [145,146]. Grape seed extract might be also used as a treatment to limit dietary fat absorption and the accumulation of fat in adipose tissue, through its activity on inhibition of the fat-metabolizing enzymes pancreatic lipase and lipoprotein lipase [147]. Additionally, intravenous and oral administration of procyanidins isolated from grape seeds resulted in significant inhibition of thrombus formation in mice models [148]. Similarly, grape seed extracts have demonstrated protection against myocardial ischaemia-reperfusion and myocardial injury in rats as well as reduction in platelet adhesion, aggregation and generation of superoxide anion [149,150,151].

Concerning oxidative stress in live cells, procyanidins from grape pomace inhibits human endothelial NADPH oxidase, the enzyme responsible for the increased production of reactive oxygen species, inhibiting ROS production at both extra- and intracellular levels [152].

In addition to the radical scavenging capacity, other valuable health promoting activities of (poly)phenols present in grape by-products has been described. Thus, concerning grape pomace and seeds, (poly)phenolic extracts have shown effective antimicrobial capacity; they are efficient against Gram-positive bacteria with medical interest (*Staphylococcus aureus*, *Bacillus cereus*, *Bacillus subtilis* and *Bacillus coagulans*), but more effective against Gram-negative bacteria such as *Escherichia coli* or *Pseudomonas aeruginosa* [153]. Similarly, grape pomace extracts have also shown antifungal activity against *Botrytis cinerea* [154].

Based on the experiments performed on the evaluation of antimicrobial activity of grape seed extracts rich in polymers of flavan-3-ols, the antimicrobial properties of grape tissue extracts has been attributed to the general mechanisms of phenolics. On this matter, Anastasiadi *et al*. (2009), demonstrated that the high concentrations of flavonoids and their derivatives in grape seeds, as well as the content in flavonoids, stilbenes, and phenolic acids in grape stems, are responsible for the antimicrobial activity of extracts from separate plant materials [155]. Furthermore, data provided by Vaquero *et al*. (2007), suggested that caffeic acid, quercetin, and quercetin-3-*O*-rutinoside are responsible for the high inhibitory capacity against *Listeria monocytogenes* [156]. In the same way, it has been demonstrated that polymeric phenolic fractions produced the highest specific inhibition activity for all *Listeria* species, but not for other pathological bacteria, such as *Bacillus cereus*, *Escherichia coli*, *Staphylococcus aureus* or *Yersinia enterocolitica* [157]. More work in this field has reported that the red grape seed extract inhibited the growth of *Candida albicans*, *Escherichia coli*, *Salmonella typhimurium*, and *Listeria monocytogenes* [158,159,160]. The study of Paulo *et al*. (2010), verified the antibacterial activity of grape phenolics attributing the antimicrobial effect to bacteriostatic action by changes in cell morphology and DNA content [161]. Therefore, the cell cycle is affected by grape phenolics. In addition to the direct antimicrobial activity by direct interaction, the cytotoxic effect of extracts of vinification waste residues against bacteria of medical interest *in vivo* would be due to immunologic induction by such polyphenols [162].

In addition to the mentioned health attributions as antioxidant and antimicrobial agents, phenolic compounds have been also pointed out as interesting bioactives for cardiovascular diseases by means of the inhibition of plasma platelet aggregation and cyclooxygenase activity, the suppression of histamine release and slow-reacting substances of anaphylaxis biosynthesis *in vitro*, a potent nitric oxide radical scavenging activity, and anti-inflammatory activities [88].

Moreover, grape seeds extracts and their constituents have been proven as inflammatory-preventive with protective effects on chemical-induced ulcerative colitis in rats [163,164,165,166]. This study evaluated the protective role of dietary grape seeds extracts on inflammatory bowel disease, attributed to their capacity to modulate gut microflora, decreasing *Faecalibacterium prausnitzii* within the intestinal lumen and blocking gut inflammatory response, indicating the proanthocyanidins in grape seeds extracts as the key components for the observed beneficial effects.

Additionally, chronic inflammation causes oxidative stress in the affected tissues, and in turn, elevated oxidative stress enhances the inflammatory response by activating redox-sensitive nuclear transcription factors [167]. Dietary antioxidants may provide a cost-effective strategy to promote health through minimizing the severity of oxidative stress and chronic inflammation. On this matter, animal and human clinical trials further demonstrated an improvement of systemic inflammation after antioxidant supplementation [168,169]. Since grape pomace contains significant amounts of antioxidants anthocyanins, catechin, epicatechin, quercetin and a few phenolic acids, this material has been suggested as beneficial for the prevention of oxidative stress and inflammatory conditions. This has been further supported at preclinical level by dietary supplementation with 250 mg grape pomace/kg per day for 12 weeks, showing significant anti-inflammatory effects in obese mice [170].

It has also been shown that grape seed extracts can provide a modulatory effect on age-related oxidative DNA damage and lipid peroxidation concerning the central nervous system in rats [171,172]. The health effect of dietary supplementation with this vinification residue was demonstrated by improved memory performance, reduced reactive oxygen species production, decreased protein carbonyl level, increased thiol level, and reduced hypoxic ischaemic brain injury in central nervous systems. In this regard, additional studies have shown that resveratrol could exert neuroprotection against ischaemia, seizure, and neurodegenerative diseases [173].

## 5. Current Extractive Techniques for Bioactive Phytochemicals of Industrial By-Products: Limitations and Possibilities to Improve

The suitability of the separate extractive techniques constitutes a critical issue to gain rational valorization of winemaking by-products. Thus, the qualitative and quantitative composition of the (poly)phenolics extracts from winery by-products depend not only on the source of the plant material concerning the cultivar, the agro-climatic conditions of the production area, and the tissue considered (leaves, stems, seeds, peels, *etc*.), but also on the physico-chemical conditions of the industrial process, as well as the combination of solvents and the extraction procedures employed. Additional factors such as the quality of the plant material, the storage conditions and its pretreatment are all decisive to define the biochemical profile of the phenolic extracts and, therefore, for the potential biological activity. Hence, all these parameters should be taken into account, in order to produce high quality extracts with valuable biological activities, suitable to be used by food, cosmetic and/or pharmaceutical industries.

Given the final purpose of the valorization of vinification residues based on the functional properties of the bioactive compounds isolated, it is necessary to purify the extract obtained, removing all the inert and undesirable components that could interfere with the final biological activity (radical scavenging, antimicrobial activity, and so on) of the extracts. This procedure will allow reduction of its odour, taste, and colour as much as possible to avoid interference with the technological processes [49]. This approach supports that organic residues (stems, seeds, peels and/or pomace) derived from the vinification process are studied to gain the most effective extraction procedures focused on minimizing the extraction time, reducing organic solvent consumption, and maintaining the recovery of compounds of interest.

To date, there exists a panel of extraction techniques to isolate phenolic compounds from the wine-making residues. The most widespread technique used to extract the (poly)phenolics from winery residues is the solvent solid/liquid extraction (SLE), even though additional procedures, namely supercritical fluids extraction (SFE), ultrasound assisted extractions (UAE), microwave assisted extractions (MAE), and high pressure and temperature extraction (HPTE) have been also assayed providing successful outcomes on phenolic extraction [174].

Regarding SLE methods, a myriad of factors have been described as involved in their efficiency, such as the type of solvent, relative solvents proportion, solvent/sample ratio, particle size, type of phenols under study, temperature, and extraction time, among others, and, therefore, there is not a single optimal extractive condition or procedure for all the types of residues derived from this agro-industrial activity.

Thus, the comparison of the suitability of the distinct methods assayed so far shows that hydroalcoholic solutions provides quite high yield of total extracts, although they are not highly selective for the distinct classes of phenolics present in the residues [52,175,176,177]. Another solvent widely employed in the extraction of winery waste phenolics is methanol (MeOH), which obtains high concentrations of these target analytes. However, the toxicity associated to this solvent restricts its use to analytical procedures. In this regard, ethanol (EtOH) has been studied as a more environmentally friendly solvent, recognized as safe according to the European Food Safety Authority (EFSA) and FAO/WHO Expert Committee on Food Additives [178,179]. Concerning the comparison between distinct solvents, recently, the effect of solvent systems (MeOH and EtOH) on the efficiency of the extraction of total phenolic compounds, total flavonoids, and individual phenolics, as well as on the antioxidant capacity of the extracts obtained from grape pomace, and its constituents (peduncle, skin and seed) has been further investigated [175]. This analysis described the close relation between the solvent used and the specific residue considered in order to get the highest yield of different compounds. This work showed MeOH as the solvent with the highest extracting capacity (Table 8). On this matter, Lapornik *et al*. [177] reported that ethanol/water and methanol/water extracts (both at ratio 7:3, *v*/*v*) of grape pomace had seven times higher values than water extracts, with 12 h generally being the appropriate time of extraction.

The wide variety of physical properties involved with the distinct organic matter resulting from vinification processes has prompted optimization of extraction procedures using alternative solvents. Hence, when considering winery derived materials with high content in lipids, the use of ethyl acetate (EtAc) demonstrated the highest efficiency because of its low polarity [101,160,180]. Furthermore, the low boiling point of EtAc facilitates its removal and reuse, and any possible residue is scarcely toxic at the remaining concentration [101].

Other critical factors affecting the extraction of phenolic compounds is the extraction time. In this regard, Bonilla *et al*. (1999) studied the extraction efficiency at four different times 5, 10, 20 and 30, in crushed and uncrushed grape pomace with EtAc and water solvents [101]. As a result, crushing the grape pomace favored the extraction of ratio for both solvents. The anthocyanins are not EtAc-soluble, allowing the separation of colorants (in the aqueous phase) from other phenols (organic phase). Concerning extraction time, 10 min for the organic extracts and 20 min for the aqueous extracts of crushed pomace were adopted as the optimum settings. The organic phase was found to contain high amounts of phenols, which have antioxidant lipid activity reasonably lower than synthetic additives. However, the dried phenols extract could be used at higher levels, thereby increasing their antioxidant effectiveness as well as biological activity with beneficial results for human health. Bucic-Kojic *et al*. optimized the yield and kinetics of the phenolics extraction for grape seeds using Peleg’s mathematical model, definitely influenced by temperature, particle size and solid–liquid ratio, with extraction time not being an important parameter after 40 min of treatment [181].

**Table 8:** Polyphenol extraction techniques and solvents for industrial application of wine-industry by-products.

Extractive Procedure	Vinification Residue	Effective Solvent	Target Compound/Assay	Ref.
Solvent extraction	Grape pomace	EtOH/Water (6:4, *v*/*v*)	Total phenolic compounds	[175]
		EtOH/Water (7:3, *v*/*v*)	Total flavonoid compounds	[175]
		Ethyl acetate/Water (1:1, *v*/*v*)	Anthocyanins (aqueous phase) and phenolics (organic phase)	[100]
		Ethyl acetate/water (9:1, *v*/*v*)	Total phenolic compounds	[176]
		MeOH/water (7:3, *v*/*v*) and EtOH/water (7:3, *v*/*v*)	Total phenolic compounds	[177]
	Grape seeds	MeOH/Water/Acetone (3:3.5:3.5, *v*/*v*/*v*)	Total phenolic compounds, total flavonoids, quercetin-3-rutinoside, myricetin	[175]
		MeOH/Water (7:3, *v*/*v*)	Catechin, epicatechin	[175]
	Grape skins	MeOH/Water/Acetone (3:3.5:3.5, *v*/*v*/*v*)	Total phenolic compounds, total flavonoids, myricetin	[175]
	Grape skins	MeOH/Ethyl acetate (1:1, *v*/*v)*	Resveratrol	[121]
		MeOH/Water (7:3, *v*/*v*)	Quercetin and quercetin-3-rutinoside	[175]
	Grape peduncules	MeOH/Water/Acetone (3:3.5:3.5, *v*/*v*/*v*)	Quercetin	[175]
		MeOH/Water (7:3, *v*/*v*)	Quercetin-3-rutinoside, myricetin, catechin	[175]
		MeOH/Water (6:4, *v*/*v*)	Total phenolic compounds, total flavonoids	[175]
	Grape stems	EtOH/Water (6:6, *v*/*v*)	Flavones	[52]
		EtOH/Water (6:4, *v*/*v*)	Flavonols	[52]
		EtOH/Water (4.5:5.5, *v*/*v*)	Proanthocyanidins	[52]
	Grape pomace/stems	EtOH/Water (9:1, *v*/*v*)	Total phenolic compounds	[176]
Solvent extraction and supercritical fluid extraction	Grape pomace	Ethyl acetate and SFE CO_2_	Total phenolic compounds	[49]
	Grape seeds	EtOH and SFE CO_2_	Total phenolic compounds	[182]
Microwave assisted exraction	Grape seeds	70 W, MeOH	Quercetin, Catechin	[158]
	Grape skins	500 W, MeOH:water (6:4, *v/v*)	Anthocyanins	[183]
Ultrasound assisted extraction	Grape skins	35 KHz, MeOH/HCl (99:1, *v/v*)	Anthocyanins, flavan-3-ols, and flavonols	[167]
		35 KHz, EtOH/Water (1:1, *v/v*)	Anthocyanins, total phenolic compounds	[46]
High pressure and temperature extraction	Grape seeds	Reactor (350 °C/200 bar)	Gallic acid, hydroxytyrosol, vanillic acid, syringic acid, and *trans*-resveratrol	[184]
Aqueous β-cyclodextrins	Grape pomace	2.5% (*w/v*) aqueous β-CD solutions	Individuals flavonols, flavan-3-ols, stilbenes, and *ortho*-diohenols	[109]

The effectiveness of the solvent not only depends on the physical properties of the residue considered, but also on the target phenolic to be extracted. Thus, the extraction of bioactive phenolics present in grape seeds using EtAc as solvent, showed that this solvent largely recover flavonols, whereas methanol has been reported to be the best solvent for extracting flavan-3-ols (catechin, epicatechin, and epillocatechin). The use of ethyl acetate or acetone with addition of water resulted in a significantly increased yield of extracted proanthocyanidins. This is a consequence of a low permeability of the sample tissues to only EtAc or MeOH, which is a non-polar aprotic solvent [184].

Even though the most evaluated winery residue is grape pomace, further analyses on other organic material from this industrial activity have pointed out the relevance of less studied materials as interesting sources of bioactive compounds. Thus, Spigno *et al*. showed grape stems as valuable source of antioxidant phytochemicals, showing a high purity of phenolic extracts from grape stems by using EtAc [185]. The use of different combinations of MeOH, EtOH and acetone (C_3_H_6_O) with water, generally, yields a significant co-extraction of concomitant substances, which decreases the final content in target bioactives in the extract, and complicates the purification.

Again, time and temperature highly influenced the success of the extraction procedure. When considering these factors, the best results were obtained by increasing temperature up to 60 °C, because at this temperature the solubility of solutes and diffusion coefficient increases without decreasing the phenolics’ stability. Nevertheless, temperature cannot be considered as an isolated factor, being closely linked to the extraction time (8 h) as a factor that should be controlled to upgrade processes from the bench to the industry [185]. In this sense, a number of works on the optimization of the panel of factors influencing the efficiency of the extraction process of (poly)phenolic compounds from winery residues based on the minimization of the experimental conditions as a time-saving approach, provided by the response surface methodology has recently been reported [52,186].

Furthermore, supercritical fluid extraction (SFE) has been pointed out as an environmentally friendly alternative to extract polyphenols since it avoids the use of large amounts of toxic solvents. In this regard, supercritical CO_2_ working at 150 bar and 30–40 °C has been employed for obtaining primary extract using ethyl acetate as extraction solvent, in order to improve its properties (high antioxidant activity, lighter colour, and no odour) without causing any thermal or chemical degradation, as CO_2_ is an inert, non-toxic, and volatile solvent. This extraction technique obtains high added-value products under moderate conditions and equipment capacity [185].

Concerning ultrasound and microwave assisted extractions (UAE and MAE), they are recognized as outstanding energy sources to promote extraction, cutting down extraction times and increasing yields as well as the quality of the extract in terms of phenolic composition and biological activity. The UAE and MAE are chiefly exploited at laboratory scale and both have already been addressed toward industrial applications [15]. When comparing these extraction methods with conventional ones, the UAE appears to be much faster than traditional procedures, such as SLE, because UAE provides a higher contact-surface area between solid and liquid phase as a consequence of the fine reduction of particle size. Moreover, acoustic cavitation of power US (frequencies range 18–40 kHz), causes cell wall disruption, enhancing mass transfer and facilitating solvent access to the cell content [187]. On the other hand, the MAE employs the microwave energy to heat polar solvents in contact with organic residues from the winery industry (grape seeds, grape skins, and so on), reducing both extraction time and solvent consumption. The MAE are performed at high temperatures (110–150 °C), which constitutes a critical feature to handle antioxidants concerning to degradative conditions [188]. The comparison of the data available in the bibliography on the (poly)phenolic extraction from grape pomace by SLE, MAE, UAE, and high pressure and temperature extraction (HPTE) methods shows higher yields of total phenolics when using methanol rather than ethanol in SLE. Furthermore, when using methanol, HPTE yielded higher extraction of phenolics than UAE and SLE, MAE being the second most effective technique. These reports suggested the critical role of the extraction time, since the increase of the contact time from 15 to 30 min leads to total phenolic content increase, whereas its extension up to 90 min resulted in a decrease of their extraction. Delgado-Torre, *et al*. also compared UAE, MAE, and superheated liquid extraction (SHLE) in different samples of grape stems, and showed that SHLE was the best extractive option for total phenolics [16].

Recently, aqueous β-cyclodextrins (CD) has also been studied as a tool for recovering phenolics from wine-industry by-products directed to be used as ingredients in the development of innovative added-value foods and nutraceuticals. Due to its specific structure, the CDs are able to form host-guest inclusion complexes with a wide range of phenolic compounds, increasing their water solubility, stability, bioactivity, and bioavailability. The effective extraction procedure was achieved with 2.5% (*w*/*v*) aqueous β-CDs solutions at 60 °C for 12–24 h (Table 8) [183].

Given the constraints of the industrial production as well as the seasonality of the winery by-products availability, extracts from this agro-economic activity required preservation for further use in applications and valorization processes. Thus, once extracted, bioactive compounds could be stored as dried extracts. This physical state displays advantages over liquid forms such as lower storage costs, stability of active phytochemicals, and their versatility to be integrated in further processes in the required concentration. On this matter, one of the drying techniques most widely used by the food industry is the spray-drying, which was optimized by Pérez-Serradilla *et al*. 2011, allowing antioxidant wine lees extracts to be obtained in powder form with similar physicochemical characteristics than those of the grape seed extracts [61].

All data recorded on the beneficial properties of extracts from winery residues are receiving attention not only from the scientific community but also from sectorial industries, involved in the valorization of resources of bioactive compounds, in order to gain further insight into the exploitation of these multipurpose materials concerning cosmetic, pharmaceutical, and dietary supplement uses. This trend has allowed the development of patents, such as for wine waste extracts for reducing plasma triglycerides, platelet aggregation, and oxidative capacity [189].

Additional uses of these under-profit materials are supported by the beverage industries. Thus, dried grape skins may be interesting ingredients for teas and other health promoting beverages (for example, isotonic drinks) since grapes are very aromatic fruits with wide flavour properties [190,191].

## 6. Conclusions

Consumption of grapes and grape-based foods is recognized as a major contributor to the beneficial effects of the “Mediterranean Diet”. Currently, grapes and their derivatives have attracted scientific interest to confirm their applications in the development of innovative added-value products. A relevant proportion of grapes (up to 15% of yields) is directed to the winery industry, which is linked to the production of high amounts of undesirable by-products, which include seeds, peels, and grape stems. These have been also evaluated for their content in bioactive compounds revealing that several factors, such as cultivar, agro-climatic conditions and degree of ripening, among others, are responsible for the wide variations in their phytochemical profiles. Accurate data on the biological functions of these compounds available nowadays have allowed their identification as responsible agents for multiple benefits involved in the prevention of degenerative processes through their integration into functional foods, nutraceuticals, and cosmetics. Hence, the most relevant activities attributed to bioactive phytochemicals from winery by-products are antioxidant, antimicrobial, anti-inflammatory, and anticancer [41,81,157,172]. Even though vinification residues are considered in general, a good source of bioactive compounds, the total amount of phenolics, as well as the specific profile concerning compound identity and their relative proportion, is strongly dependent on the type of residue considered. In this regard, the identification of the specific (poly)phenols of the separate winery wastes has prompted the development of more extensive panels of valorization options for these residues. Thus, recycling of winery co-products or side streams constitutes an opportunity for providing valuable materials to pharmaceutical, cosmetic, nutraceutical, and food industries, contributing to reduced costs and environmental impact linked to the disposal of these by-products in the production areas.

## 7. Future Prospects

Over the last decades a large knowledge base on the bioactivity, bioavailabily and toxicology of phytochemicals has emerged. This has allowed the relevance of bioactive compounds as health promoters in the hallmark of healthy diets and lifestyles to be enhanced. To gain knowledge on the potential of bioactives to reduce the incidence and/or severity of degenerative diseases, their interaction within their food matrices related to the dietary administration as well as the specific bioavailability in pharmacological or food formulas needs studies under *in vitro* and *in vivo* conditions. Winery by-products are good sources of phytochemicals and natural antioxidants, and partially exploited for their health promoting properties mainly in the cosmetic industry. However, even though some applications of these agro-industrial by-products have been found to date, their valorization needs additional commitment concerning the development of novel extraction, isolation, purification, and recovery procedures to obtain higher amounts of healthy bioactive phytochemicals and to deliver standardized potency in different fields. Efforts should also be focused on the isolation and structural elucidation of novel grape phenolic compounds from the less characterized materials derived from vinification processes (grape stems), using chromatographic and spectroscopic tools. In this sense, adequate procedures concerning the target product and time-saving technologies to allow the integration of the obtained results for sectorial industries, will provide successful achievements for phytochemical recovery and innovative products, which could contribute to the reduction of environmental pressure in winery areas from winery by-products, and promote the application of isolated bioactives in feed, food, pharmaceutical, and cosmetic industries.

## Abbreviations

BHT: Buthylated hydroxytoluene
BHA: Buthylated hydroxyanisole
Cy: Cyanidin
Del: Delphinidin
dw: dry weight
fw: fresh weight
GAE: Galic acid equivalents
Gal: Galactoside
Glc: Glucoside
Gluc: Glucuronide
HDL: High-density lipoprotein
HPLC–DAD: High Performance Liquid Chromatography–Diode Array Detector
HPLC–FD: High Performance Liquid Chromatography–Fluorescence Detector
HPLC–UV: High Performance Liquid Chromatography–Ultraviolet Detector
HPTE: High pressure and temperature extraction
Isrhm: Isorhamntin
LDL: Low-density lipoprotein
K: Kaempferol
MAE: Microwaves assisted extractions
Mv: Malvidin
Myr: Myricetin
Pn: Peonidin
Q: Quercetin
Res: Resveratrol
Rha: Rhamnoside
Rut: Rutinoside
ROS: Reactive Oxygen Species
SFE: Supercritical fluids extraction
SLE: Solvent extraction
TBHQ: *tert*-buthylhydroxyquinone
UAE: Ultrasound assisted extractions
Xyl: Xyloside
